# Large-scale proteomic analysis of human brain identifies proteins associated with cognitive trajectory in advanced age

**DOI:** 10.1038/s41467-019-09613-z

**Published:** 2019-04-08

**Authors:** Aliza P. Wingo, Eric B. Dammer, Michael S. Breen, Benjamin A. Logsdon, Duc M. Duong, Juan C. Troncosco, Madhav Thambisetty, Thomas G. Beach, Geidy E. Serrano, Eric M. Reiman, Richard J. Caselli, James J. Lah, Nicholas T. Seyfried, Allan I. Levey, Thomas S. Wingo

**Affiliations:** 1 Division of Mental Health, Atlanta VA Medical Center, Decatur, GA 30033 USA; 2 Department of Psychiatry, Emory University School of Medicine, Atlanta, GA 30322 USA; 3 Department of Biochemistry, Emory University School of Medicine, Atlanta, GA 30322 USA; 4 Department of Psychiatry, Icahn School of Medicine at Mount Sinai, New York, NY 10029 USA; 5 Department of Genetic and Genomic Sciences, Icahn School of Medicine at Mount Sinai, New York, NY 10029 USA; 6 Seaver Autism Center for Research and Treatment, Icahn School of Medicine at Mount Sinai, New York, NY 10029 USA; 7 Sage Bionetworks, Seattle, WA 98109 USA; 8 Johns Hopkins School of Medicine, Baltimore, MD 21205 USA; 9 Unit of Clinical and Translational Neuroscience, Laboratory of Behavioral Neuroscience, National Institute on Aging, National Institutes of Health, Bethesda, MD 20892 USA; 10 Banner Sun Health Research Institute, Sun City, AZ 85351 USA; 11 Banner Alzheimer’s Institute, Arizona State University and University of Arizona, Phoenix, AZ 85351 USA; 12 Department of Neurology, Mayo Clinic, Scottsdale, AZ 85259 USA; 13 Department of Neurology, Emory University School of Medicine, Atlanta, GA 30322 USA; 14 Division of Neurology, Atlanta VA Medical Center, Decatur, GA 30033 USA; 15 Department of Human Genetics, Emory University School of Medicine, Atlanta, GA 30322 USA

## Abstract

In advanced age, some individuals maintain a stable cognitive trajectory while others experience a rapid decline. Such variation in cognitive trajectory is only partially explained by traditional neurodegenerative pathologies. Hence, to identify new processes underlying variation in cognitive trajectory, we perform an unbiased proteome-wide association study of cognitive trajectory in a discovery (*n* = 104) and replication cohort (*n* = 39) of initially cognitively unimpaired, longitudinally assessed older-adult brain donors. We find 579 proteins associated with cognitive trajectory after meta-analysis. Notably, we present evidence for increased neuronal mitochondrial activities in cognitive stability regardless of the burden of traditional neuropathologies. Furthermore, we provide additional evidence for increased synaptic abundance and decreased inflammation and apoptosis in cognitive stability. Importantly, we nominate proteins associated with cognitive trajectory, particularly the 38 proteins that act independently of neuropathologies and are also hub proteins of protein co-expression networks, as promising targets for future mechanistic studies of cognitive trajectory.

## Abstract (web summary)

Cognitive abilities tend to decline over time in advanced age, yet some individuals experience stable abilities or rapid decline. Here the authors present a proteome-wide association study of cognitive trajectory, and identify 38 proteins associated with cognitive resilience.

## Introduction

In advanced age, trajectory of cognitive performance over time can range from stability to rapid decline. Like all complex human traits, cognitive trajectory varies from person to person and is determined by a combination of genetic and environmental factors including premorbid cognitive abilities, educational attainment, lifestyle choices, and environmental exposures^1,2^. A stable cognitive trajectory in advanced age is desirable and may reflect cognitive resilience, while decline in cognitive performance may be the earliest manifestation of a serious neurodegenerative disease^1,3^. Cognitive decline may ultimately lead to a diagnosis of mild cognitive impairment (MCI) or dementia. Hence, variation in cognitive trajectory can influence onset age for MCI or dementia.

Studying individual cognitive trajectory is strategic for several reasons. First, it captures all the factors affecting cognition, including diverse pathologies as well as biological mechanisms independent of pathologies^4–6^. Second, it likely captures co-occurring disease processes and co-occurring age-related pathologies, which are known to be prevalent in the brains of aged individuals^4,5^. Thus, it provides more comprehensive information than the traditional case/control status based on clinical or pathological diagnosis. Third, cognitive trajectory is estimated from periodically and prospectively assessed cognitive performance. Such trajectory captures the progressive nature of cognitive decline, from the asymptomatic stage to the manifestation of mild cognitive impairment or dementia, and thus is more germane to early intervention and prevention. Fourth, as will be shown, within each category of pathology or clinical diagnosis of cognitive status, different persons have different cognitive trajectories making cognitive trajectory relevant to individuals with or without dementia.

Despite variation in cognitive trajectory, in advanced age the majority of individuals tend to experience cognitive decline. Cognitive decline has been suggested to be attributable to dementia pathologies. However, pathologies such as β-amyloid plaques, neurofibrillary tangles, microinfarct, macroinfarct, and Lewy bodies together capture only about 41% of the variance in cognitive trajectory, leaving 59% unexplained^5,7^. It is unclear what causes the large majority of the variance in cognitive trajectory. Proteins, as functional gene products, are well-suited for studying biological processes underlying individual variation in cognitive trajectory. Yet, no proteome-wide study of cognitive trajectory has been performed to date.

**Fig. 1:**
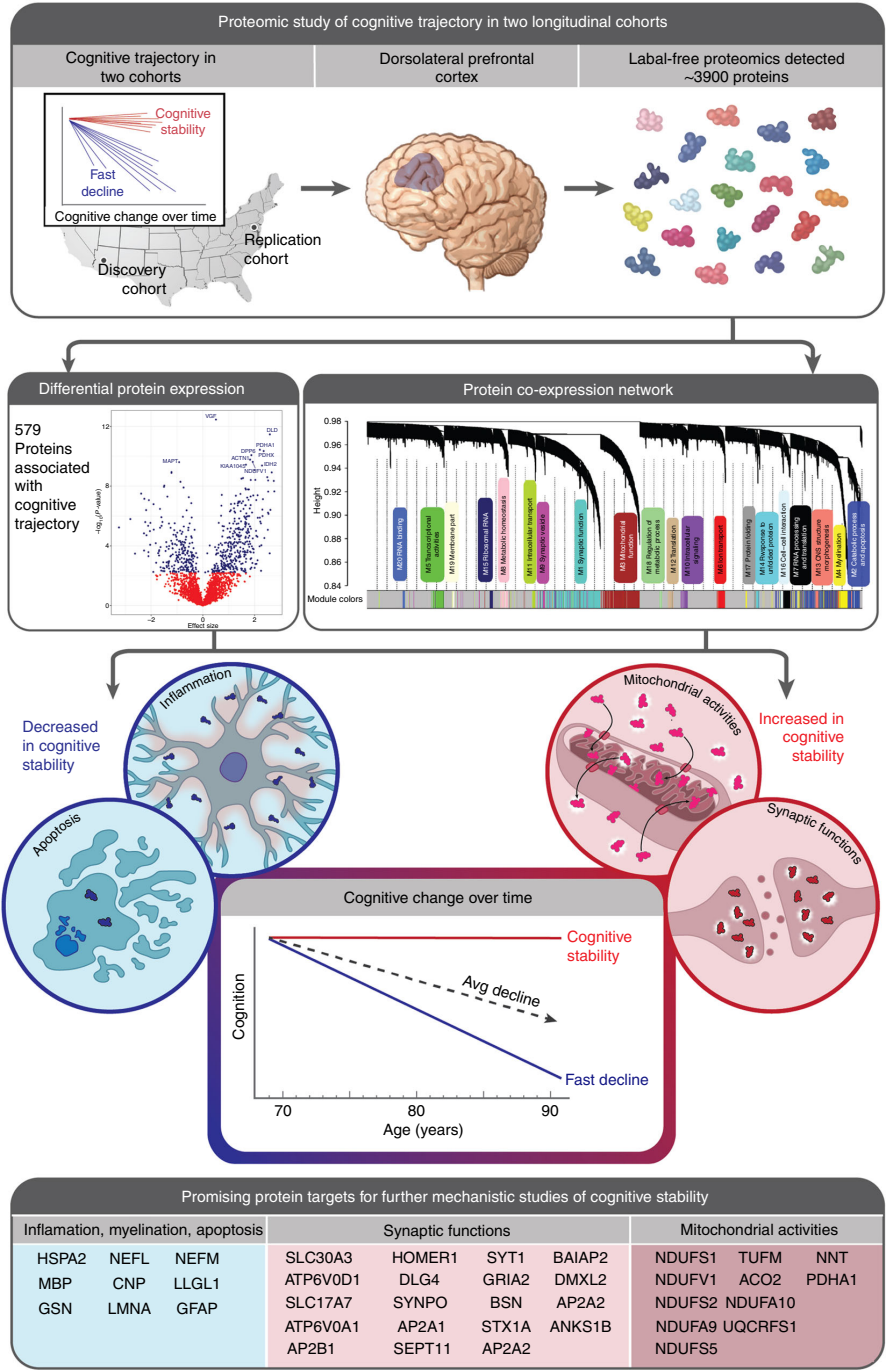
Overview of the study design and results. The samples used in this study were from participants in two large prospective studies of aging in the United States who donated their brains upon death. Each subject in the present study were initially cognitively normal and had antecedent cognitive data used to estimate personal cognitive trajectories. Whole brain proteomic analysis of dorsolateral prefrontal cortex was used to estimate protein abundance for each participant. Proteome-wide association study of cognitive trajectory and protein co-expression network analysis revealed that individuals with cognitive stability (i.e., relatively little decline in cognition over time) have decreased abundance of the proteins involved in inflammation and apoptosis but increased abundance of the proteins involved in mitochondrial activities and synaptic function. Promising protein targets for further study are presented

To identify biological processes underlying cognitive trajectory, we here perform an unbiased, large-scale proteome-wide association study of cognitive trajectory using a discovery and replication cohort of initially cognitively unimpaired, longitudinally assessed older-adult brain donors (Fig. 1). We then perform gene ontology enrichment analysis on significantly associated proteins to glean a deeper understanding of the biological processes underlying individual differences in cognitive trajectory. Next, we determine which proteins associated with cognitive trajectory are key drivers of protein co-expression networks because these proteins are likely promising targets for future mechanistic studies of cognitive trajectory. Lastly, we determine the proteins that are associated with cognitive trajectory independently of the traditional Alzheimer’s disease pathologies (Fig. 1). Together, our findings establish a framework for understanding the molecular mechanisms underlying individual differences in cognitive trajectory.

## Results

### Subjects

The Banner discovery cohort consisted of 104 participants who were non-demented at baseline and were followed for up to 14 years (Table 1). Among these, 48% were women, 27% had the final clinical diagnosis of AD, 73% had the final diagnosis of normal control, and median age at death was 87 (Table 1). The BLSA replication cohort had 39 participants who were followed for up to 20 years (Table 1). Of these, 39% were women, 41% had a final clinical diagnosis of AD and 59% had a final clinical diagnosis of normal control, and median age at death was 91 (Table 1).

**Table 1:** Demographic and clinical characteristics of the discovery and replication cohorts

Characteristic	Banner *N* = 104	BLSA *N* = 39
*Age at enrollment*		
Mean ± SD	80.7 ± 6.6	74.8 ± 6.9
Median; Range	80.6; [61.5–96.4]	75.3; [58.5–89.3]
*Age at death*		
Mean ± SD	87.0 ± 6.7	87.2 ± 7.7
Median; range	87; [74–103]	91; [71–99]
Sex (% female, *N*)	48.1% (50)	38.5% (15)
*Education (years*)		
Mean ± SD	14.6 ± 2.6	16.6 ± 3.0
Median; Range	16; [8.0–20.0]	18; [8.0–20.0]
*Clinical diagnosis*		
AD (%, N)	26.9% (28)	41.0% (16)
Control (%, N)	73.1% (76)	59.0% (23)
*Follow-up duration (years)*		
Mean ± SD	5.1 ± 3.2	9.3 ± 3.9
Median; Range	4.4; [0.5–14.0]	8.5; [3.9–20.4]
*PMI (h)*		
Mean ± SD	2.9 ± 0.9	13.8 ± 5.5
Median; Range	2.7; [1.2–5.5]	14.5; [2.0–24.0]
*Braak stage (%, N)*		
I	4.8% (5)	0% (0)
II	10.6% (11)	15.4% (6)
III	25.0% (26)	15.4% (6)
IV	39.4% (41)	33.3% (13)
V	14.4% (15)	10.3% (4)
VI	5.8% (6)	25.6% (10)
CERAD stage	Criteria not met: 12.5% (13)	0 (none): 20.5% (8)
	Definite AD: 25.0% (26)	A (sparse): 7.7% (3)
	No AD: 28.8% (30)	B (moderate): 30.8% (12)
	Possible AD: 31.7% (33)	C (frequent): 41.0% (16)
	Probable AD: 1.9% (2)	
*Slope of cognitive trajectory (MMSE/year)*		
*All participants*		
Mean ± SD	−0.5 ± 0.8	−0.4 ± 0.6
Median; Range	−0.3; [−4.9 to 0.2]	−0.1; [−2.4 to 0.1]
*By clinical diagnosis (median; range)*		
Controls	−0.1; [−1.6 to 0.2]	−0.1; [−0.7 to 0.1]
AD cases	−1.1; [−4.9 to −0.1]	−0.4; [−2.4 to 0.0]
*By Braak stage (median; range)*		
I and II	−0.1; [−0.9 to 0.2]	−0.2; [−0.7 to 0.0]
III and IV	−0.2; [−2.1 to 0.1]	0.0; [−2.4 to 0.1]
V and VI	−1.1; [−4.9 to −0.1]	−0.4; [−1.8 to 0.0]

The table summarizes the demographic and clinical characteristics of the Banner (discovery) and BLSA (replication) cohorts. The following terms Alzheimer’s disease (AD), post-mortem interval (PMI), Mini-Mental State Exam (MMSE), and Consortium to Establish a Registry for Alzheimer’s Disease (CERAD) are used as acronyms in the table. The underlying data are available through the Synapse platform (https://www.synapse.org/#!Synapse:syn7170616 and https://www.synapse.org/#!Synapse:syn3606086) and Supplementary Data 1

### Personalized cognitive trajectories

**Fig. 2:**
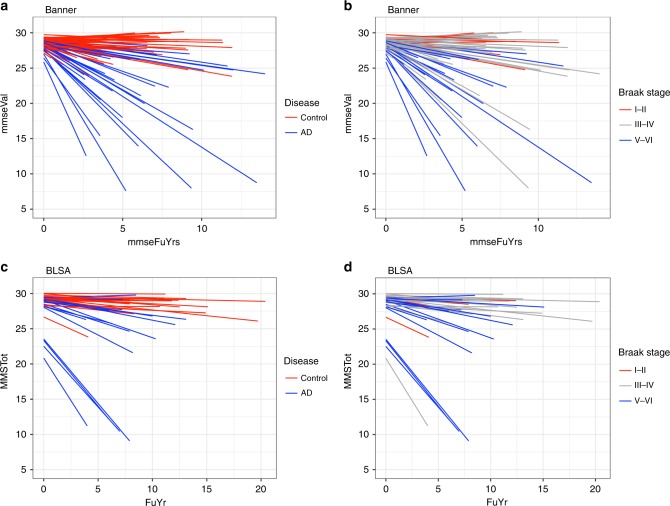
Person-specific cognitive trajectory. This figure summarizes cognitive trajectory for each individual with colors indicating clinical diagnosis and Braak stage. Individual cognitive trajectory in the Banner cohort by **a** last clinical diagnosis (AD or control) prior to death and **b** Braak stage. Individual cognitive trajectory in the BLSA cohort by **c** clinical diagnosis; **d** Braak stage. A positive slope or small negative slope reflects cognitive stability. A larger negative slope reflects faster cognitive decline. The underlying data are available through the Synapse platform (protein abundance, https://www.synapse.org/#!Synapse:syn7170616 and https://www.synapse.org/#!Synapse:syn3606086) and Supplementary Data 1

The individual cognitive trajectory was estimated for each participant in both cohorts (Fig. 2; Table 1; Supplementary Data 1). Cognitive trajectories by last clinical diagnosis before death and by Braak stage were depicted in Fig. 2. Trajectories with slopes at or near zero reflect cognitive stability, while trajectories with large negative slopes indicate faster cognitive decline. We depicted cognitive trajectory by Braak stage for demonstration here because neurofibrillary tangles have been suggested to account for more cognitive decline than β-amyloid plaques^8^. The median rate of cognitive change was −0.3 unit of MMSE score per year for Banner participants and −0.1 unit of MMSE per year for BLSA participants (Table 1). Analyzed jointly, the Spearman’s rank correlation between cognitive trajectory and clinical diagnosis of cognitive status was −0.49 (95% confidence interval [CI] of −0.60 to −0.35, *p*-value of 4.3 × 10^–10^) and between cognitive trajectory and Braak stage was −0.39 (95% CI of −0.51 to −0.24, *p*-value of 1.5 × 10^–6^). These data reveal that (i) cognitive trajectory varied within a given clinical diagnosis or Braak stage; (ii) faster cognitive decline was not exclusively seen in AD cases or in brains with greater Braak stages; and, (iii) cognitive trajectory provides complementary information to the clinical diagnosis and neuropathology findings.

### Proteome-wide association study of cognitive trajectory

After label-free quantification, 3710 proteins in Banner cohort and 3933 proteins in BLSA cohort were detected with 10% or fewer missing values. Among the detected proteins, 2752 protein isoforms were detected in both Banner and BLSA cohorts. In the Banner discovery cohort, 354 proteins were associated with cognitive trajectory at FDR < 0.05 after adjusting for sex, age at enrollment, education, age at death, PMI, and batch (*N* = 104, Supplementary Data 2; Supplementary Fig. 1A). The top five proteins were VGF, SEPT5, DBI, MAPT, and KIAA1045 (aka PHF24). In the BLSA replication cohort, 127 proteins were associated with cognitive trajectory at FDR < 0.05 after adjusting for sex, age at enrollment, education, age at death, and PMI (*N* = 39; Supplementary Data 2, Supplementary Fig. 1B). The top 5 proteins were DLD, ABHD10, VDAC1, NDUFV1, and PDHB. Likely reasons for difference in the top differentially expressed proteins in the two cohorts are the differences in demographic composition, follow-up duration, range of cognitive trajectory, and false discovery. Given these differences, a meta-analysis of these data was performed to enhance true positive findings, as the meta-analysis is designed to integrate the results of several independent studies and provide a more precise estimate of the association between predictor and outcome than any individual study contributing to the pooled analysis^9^.

**Fig. 3:**
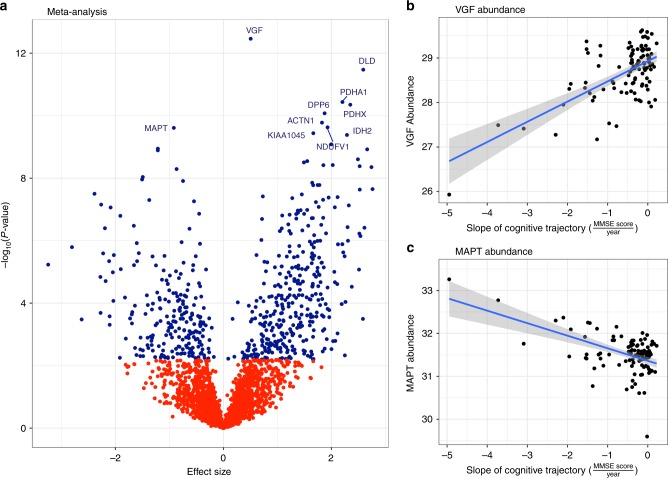
Differential protein expression in cognitive trajectory. This figure summarizes the meta-analysis of the proteome-wide association study of cognitive trajectory in the discovery (Banner) and replication (BLSA) cohort. **a** Volcano plot for the meta-analysis of the proteome-wide association studies of cognitive trajectory in the discovery and replication cohorts. A total of 579 proteins were associated with cognitive trajectory at FDR < 0.05. Among these, 350 proteins had increased abundance and 229 had decreased abundance in cognitive stability. The underlying data are available as Supplementary Data 2. In **b**, **c**, protein abundance vs. slope of cognitive trajectory is plotted with the best-fit regression line drawn in blue and the 95% confidence interval for the regression line shaded in gray. **b** Higher VGF protein level was associated with cognitive stability. Of note, for cognitive trajectory, a small negative slope reflects slow decline and a large negative slope reflects fast decline. Cognitive stability is reflected by a positive slope or very small negative slope of the cognitive trajectory. **c** Lower MAPT protein level was associated with cognitive stability. The data underlying **b**, **c** are available through the Synapse platform (protein abundance, https://www.synapse.org/#!Synapse:syn7170616 and https://www.synapse.org/#!Synapse:syn3606086) and Supplementary Data 1 (cognitive trajectories)

The meta-analysis showed 579 proteins associated with cognitive trajectory in the same directions in both the discovery and replication cohorts and at FDR < 0.05 among the 2752 protein isoforms commonly detected in both cohorts (*N* = 143 individuals; Fig. 3a, Supplementary Data 2). Regarding the direction of association, 350 proteins had increased abundance while 229 had decreased abundance in cognitive stability. For instance, VGF protein had increased abundance and MAPT protein had decreased abundance in cognitive stability (Fig. 3b, c). VGF is a neuropeptide that regulates synaptic function^10^, synaptic plasticity^11^, and hippocampal memory consolidation^12^. MAPT (microtubule-associated protein tau) is involved in axonal transport, synaptic plasticity, and synaptic function^13^, and aggregation of hyperphosphorylated tau proteins can lead to the formation of neurofibrillary tangles and Alzheimer’s dementia^14^. Going forward, we will refer to proteins with increased abundance in cognitive stability as higher-abundance and proteins with decreased abundance in cognitive stability as lower-abundance proteins.

Of the 579 cognitive trajectory-associated proteins, we hypothesized that some would interact potentially as members of distinct biological processes or organelles. Thus, we asked whether there was evidence for protein–protein interaction (PPI) using experimentally validated data available on BioGRID v3.5.165^15^. The 569 unique proteins among these 579 proteins (10 had isoforms) were categorized into presynaptic (*N* = 159), postsynaptic (*N* = 150), both (present in both presynaptic and postsynaptic density; *N* = 20), or other (*N* = 240) using Boyken et. al. presynaptic list^16^ and Bayes et. al. postsynaptic density list^17^ (Supplementary Data 3). Additionally, we labeled any of these proteins as mitochondrial if they are located in mitochondria based on Uniprot subcellular location^18^. Thus, some proteins may have more than one designation. For instance, of the 157 proteins designated as mitochondrial, 112 were presynaptic, 10 postsynaptic, and 35 were other (Supplementary Data 3). For the 569 unique proteins, 484 had at least one PPI, and there were a total of 2587 PPIs among these 484 proteins (Supplementary Data 3; Supplementary Fig. 4). Focusing on PPIs emanating from presynaptic proteins, we found 374 proteins with interactions (nodes) and 1745 PPIs (edges) (Supplementary Fig. 5A). Focusing on PPIs originating from postsynaptic proteins, we found 353 proteins with interactions (nodes) and 864 PPIs (edges) (Supplementary Fig. 5B). Thus there is ample evidence that the proteins associated with cognitive trajectory interact with each other and reveal many PPIs within and between presynaptic and postsynaptic proteins associated with cognitive trajectory. Interestingly, mitochondrial proteins were featured prominently among cognitive trajectory-associated proteins, and more findings about mitochondrial proteins are followed.

### Enrichment analysis of cognitive trajectory proteins

To understand the biological processes related to the 579 proteins associated with cognitive trajectory, we performed GO enrichment analysis for the 229 proteins with lower-abundance in cognitive stability and 350 proteins with higher-abundance in cognitive stability, separately. The lower-abundance proteins were enriched for inflammatory response, apoptosis, endothelial function, and RNA processing at adjusted *p*-value < 0.05 by Fisher’s exact test (Table 2, Supplementary Data 4). The higher-abundance proteins were enriched for mitochondrial function and synaptic transmission at adjusted *p*-value < 0.05 by Fisher’s exact test (Table 2, Supplementary Data 4). Next, we mapped these 579 cognitive trajectory-associated proteins to cell types using brain cell type signatures from Sharma et al.^19^, Zeisel et al.^20^, and Damarnis et al.^21^. The lower-abundance proteins in cognitive stability were significantly enriched for signatures of oligodendrocyte, astrocyte, and microglia after multiple testing correction (Table 2, Supplementary Data 4). The higher-abundance proteins were strongly enriched for neurons and mildly enriched for astrocytes at adjusted *p*-value < 0.05 by Fisher’s exact test (Table 2, Supplementary Data 4). Together, our findings suggest that the 350 proteins with increased abundance in cognitive stability participate in mitochondrial activities and synaptic transmission in neurons.

**Table 2:** Gene ontology and cell-type enrichment analyses for cognitive trajectory-associated proteins

	Low-abundance proteins in cognitive stability	High-abundance proteins in cognitive stability
GO biological process	Inflammatory response, apoptosis, endothelial function, RNA processing	Mitochondrial function, synaptic transmission
GO cellular component	Inflammatory response, cellular adhesion	Mitochondrion, synaptic vesicle, neuron
GO molecular function	RNA binding, cell–cell adhesion	Cellular respiration, syntaxin binding
Brain cell type	Oligodendrocyte, astrocyte, microglia	Neuron, astrocyte

The table summarizes gene ontology (GO) and cell-type enrichment analysis for the 579 cognitive trajectory-associated proteins categorized as either low- or high-abundance in cognitive stability. The underlying data are provided as a Supplementary Data 4

### Proteins in cognitive trajectory adjusted for neuropathology

To determine whether the proteins associated with cognitive trajectory act through or independently of traditional AD pathologies, we performed another proteome-wide association study of cognitive trajectory adjusting for β-amyloid plaque and neurofibrillary tangle pathology in the discovery and replication cohort separately, followed by a meta-analysis. At FDR < 0.05, 57 proteins in Banner and 12 proteins in BLSA were associated with cognitive trajectory after adjusting for sex, age at enrollment, education, age at death, PMI, β-amyloid plaques, and neurofibrillary tangles (Supplementary Data 5). The meta-analysis revealed 232 proteins associated with cognitive trajectory at FDR < 0.05 in the same directions in both datasets (Supplementary Data 5).

To identify biological processes associated with these 232 proteins, GO enrichment analysis was performed on the higher-abundance and lower-abundance proteins in cognitive stability separately. Lower-abundance proteins were enriched for the processes of glycolysis, assembly of neurofilament bundle, cell–cell adhesion, and RNA binding, and for oligodendrocyte, astrocyte, and microglia (Table 3; Supplementary Data 6). Higher-abundance proteins in cognitive stability were enriched for processes of mitochondrial activities and synaptic transmission, and for neurons (Table 3; Supplementary Data 6).

**Table 3:** Gene ontology and cell type enrichment analyses of cognitive trajectory-associated proteins adjusting for neuropathology

	Low-abundance proteins in cognitive stability	High-abundance proteins in cognitive stability
GO biological process	Glycolysis, neurofilament bundle assembly	Mitochondrial activities, synaptic transmission
GO cellular component	Focal adhesion, axon, neurofibrillary tangle, microtubule	Mitochondrion, synaptic vesicle
GO molecular function	RNA binding, cell–cell adhesion	Syntaxin binding
Brain cell type	Oligodendrocyte, astrocyte, microglia	Neuron

The table summarizes gene ontology (GO) and cell-type enrichment analysis for the 232 cognitive trajectory-associated proteins adjusting for β-amyloid and neurofibrillary tangle pathology. Differentially abundant proteins are categorized as either low- or high-abundance in cognitive stability. The underlying data are provided as a Supplementary Data 6

Notably, the proteome-wide association study reveals that the proteins with increased abundance in cognitive stability were involved in mitochondrial activities and synaptic transmission in neurons independently of effects of β-amyloid plaques and neurofibrillary tangles. In addition, the proteins with decreased abundance in cognitive stability were involved in inflammatory response, apoptosis, and endothelial function in glial cells, likely acting via β-amyloid plaques and neurofibrillary tangles. In contrast to the proteins with higher-abundance in cognitive stability, adjusting for traditional neuropathology influenced those with lower-abundance in cognitive stability to a much greater degree and changed biological processes represented.

### Protein co-expression network construction

**Fig. 4:**
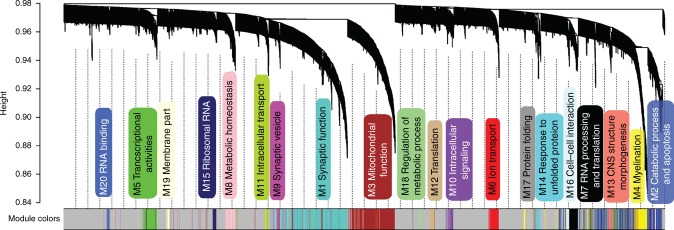
Protein co-expression network. This figure shows the WGCNA cluster dendogram and 20 distinct protein co-expressed modules defined by dendogram branch cutting in the Banner cohort. Each module is given in a box with the module number and a functional summary of the proteins in the module derived by gene ontology enrichment. The underlying data are provided as a Supplementary Data 7

Many biological functions require a coordinated effort of a large number of genes and proteins, and systems biology approaches seek to identify gene products that act in concert with one another in co-expression networks^22^. We identified 20 modules of strongly co-expressed proteins in the Banner cohort using WGCNA and then gleaned the biological processes enriched in these modules with GO enrichment analysis (Fig. 4; Supplementary Data 7). The five largest modules were enriched for synaptic function (M1, 359 proteins), catabolic process and apoptosis (M2, 296 proteins), mitochondrial function (M3, 276 proteins), myelination (M4, 141 proteins), and transcriptional activities (M5, 83 proteins) at adjusted *p* < 0.05 by Fisher’s exact test (Supplementary Data 7). Of note, our group previously performed a detailed co-expression network analysis for the BLSA cohort and identified 16 networks of co-expressed proteins^23^. We used these BLSA protein networks in our subsequent analyses.

We performed a pair-wise overlap analysis between pairs of Banner and BLSA protein co-expression modules using Fisher’s exact test. We found nine pairs of significant overlap after multiple testing correction: Banner M1 (synaptic function) with BLSA M1 (synaptic transmission, *∩* = 187 proteins, *p* = 2.53E−23, adjusted *p* = 7.40E−06) and BLSA M4 (synaptic membrane/dendrite, *∩* = 59, *p* = 3.96E−06, adjusted *p* = 0.0015); Banner M2 (catabolic process and apoptosis) with BLSA M2 (myelination, *∩* = 66, p = 4.27E−06, adjusted *p* = 0.0017) and BLSA M6 (inflammatory response, *∩* = 56, p = 2.28E−08, adjusted *p* = 1.54E−05); Banner M3 (mitochondrial function) with BLSA M3 (mitochondrial function, *∩* = 167, p = 5.51E−37, adjusted *p* = 7.40E−06); Banner M4 (myelination) with BLSA M2 (myelination, *∩* = 100, *p* = 5.96E−17, adjusted *p* = 7.40E−06), among others (Supplementary Data 7).

### Cognitive trajectory proteins and protein networks

**Fig. 5:**
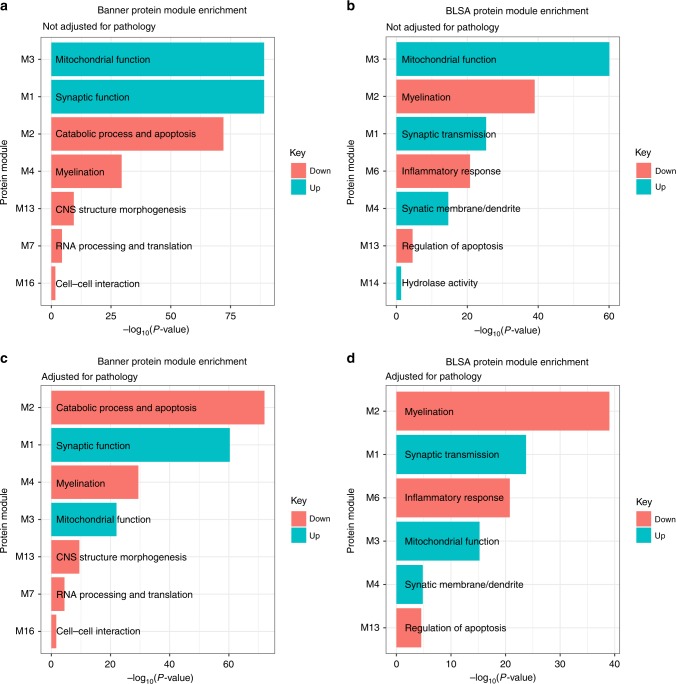
Modules enriched for cognitive trajectory-associated proteins. This figure summarizes the protein co-expression modules that are enriched for cognitive trajectory-associated proteins for each cohort with and without adjustment for neuropathologies. Enrichment was determined by Fisher’s exact test and reported *p*-values are adjusted for FDR. There are 579 cognitive trajectory-associated proteins in Banner and BLSA cohorts, which were divided into lower-abundance proteins in cognitive stability (229 proteins, colored in salmon) and higher-abundance proteins in cognitive stability (350 proteins, colored in turquoise), respectively. **a** Banner protein module enrichment; **b** BLSA protein module enrichment. After adjusting for β-amyloid plaques and neurofibrillary tangles, there were 232 cognitive trajectory-associated proteins, and enrichment was performed separately for lower-abundance proteins in cognitive stability (81 proteins, colored in salmon) and higher-abundance proteins in cognitive stability (151 proteins, colored in turquoise), respectively. **c** Banner protein module enrichment; **d** BLSA protein module enrichment. Source data are provided as a Source Data file

The 579 proteins associated with cognitive trajectory from the meta-analysis were tested for enrichment in each network of co-expressed proteins in the Banner and BLSA cohorts, respectively. We considered the 350 higher-abundance proteins and the 229 lower-abundance proteins in cognitive stability separately. The higher-abundance proteins in cognitive stability were enriched in modules involved in synaptic function (Banner M1, BLSA M1, and M4) and mitochondrial function (Banner M3 and BLSA M3) in both cohorts at adjusted *p* < 0.05 by Fisher’s exact test (Fig. 5a). On the other hand, the lower-abundance proteins in cognitive stability were enriched in the modules involved in apoptosis (Banner M2, BLSA M13), myelination (Banner M4, BLSA M2), and inflammatory response (BLSA M6 and indirectly Banner M2 by virtue of overlap) in both cohorts at adjusted *p* < 0.05 by Fisher’s exact test (Fig. 5b).

Among the 232 proteins associated with cognitive trajectory independently of β-amyloid plaques and neurofibrillary tangles, we found that the higher-abundance proteins in cognitive stability were enriched for modules of synaptic function (Banner M1, BLSA M1, and BLSA M4), and mitochondrial function (Banner M3 and BLSA M3) in both cohorts at adjusted *p* < 0.05 by Fisher’s exact test (Fig. 5c). The lower-abundance proteins in cognitive stability were enriched for the modules involving apoptosis (Banner M2, BLSA M13), myelination (Banner M4, BLSA M2), and inflammatory response (BLSA M6 and Banner M2 by virtue of overlap) in both cohorts at adjusted *p* < 0.05 by Fisher’s exact test (Fig. 5d).

We note that through both approaches—proteome-wide association study of cognitive trajectory and protein co-expression networks—we found consistent results that the over-expressed proteins in cognitive stability were enriched for mitochondrial activities and synaptic function independently of β-amyloid plaques and neurofibrillary tangles.

### Modules of co-expressed proteins and cognitive trajectory

We examined direct correlation between the modules of co-expressed proteins and cognitive trajectory in each cohort. In the Banner network, 10 of its 20 modules were associated with cognitive trajectory at FDR < 0.05 (Table 4). The top six of these 10 modules were the same modules found to be enriched among the proteins associated with cognitive trajectory (Table 4, Fig. 5a). In BLSA, four modules were associated with cognitive trajectory at by linear regression *p* < 0.05 but only one remained significantly associated with cognitive trajectory after adjusting for multiple testing (M3/mitochondrial function, *p* = 0.0025, adjusted *p* = 0.039), likely due to small sample size and thus lower power (Table 4).

**Table 4:** Correlations between modules of co-expressed proteins and cognitive trajectory

	Module	Biological process	*r*	*p*-value	Adjusted *p*-value
BANNER (*N* = 104)	M1	Synaptic function	0.52	1.52E−08	1.52E−07
	M3	Mitochondrial function	0.39	3.73E−05	0.00019
	M2	Catabolic process and apoptosis	−0.59	4.15E−11	8.30E-10
	M13	CNS structure morphogenesis	−0.41	1.45E−05	9.66E−05
	M4	Myelination	−0.34	0.000329	0.00132
	M7	RNA processing and translation	−0.32	0.000847	0.00282
	M14	Response to unfolded protein	−0.26	0.00758	0.0217
	M16	Cell–cell interaction	−0.24	0.0121	0.0270
	M6	Ion transport	−0.24	0.0144	0.0288
	M11	Intracellular transport	0.25	0.0097	0.0243
BLSA (*N* = 35)	M3	Mitochondrial function	0.50	0.0025	0.039
	M4	Synaptic membrane/dendrite	0.40	0.0180	0.085
	M6	Inflammatory response	−0.39	0.0189	0.085
	M8	Ribonucleoprotein complex	−0.39	0.0214	0.085

The table summarizes the Pearson correlation (*r*) between modules of co-expressed proteins and cognitive trajectory. The underlying data are available through the Synapse platform (https://www.synapse.org/#!Synapse:syn7170616 and https://www.synapse.org/#!Synapse:syn3606086) and Supplementary Data 1

After adjusting for β-amyloid plaques and neurofibrillary tangles by linear regression, only two of the Banner modules M1/Synaptic function (*β* = 0.039, *p* = 6.74E−06, adjust *p* = 0.00013) and M2/Catabolic processes and apoptosis (*β* = −0.032, *p* = 3.02E−05, adjust *p* = 0.00030) remained significantly associated with cognitive trajectory after multiple testing adjustment at FDR < 0.05. For BLSA, only one module, M3/mitochondrial function (*β* = −0.097, *p* = 0.0059, adjust *p* = 0.095), was associated with cognitive trajectory at adjusted *p*-value < 0.1 by linear regression after adjusting for β-amyloid plaques and neurofibrillary tangles.

### Cognitive trajectory-associated proteins and hub proteins

In protein co-expression networks, hub proteins are likely important proteins because they are highly correlated with other proteins in the module. We sought to determine whether proteins associated with cognitive trajectory were also hub proteins in the Banner and BLSA co-expression networks. Hub proteins were defined as proteins with intramodular kME in the top 90th percentile. We focused on the seven Banner and seven BLSA modules that the 579 cognitive trajectory-associated proteins were enriched in (Fig. 5). In Banner, these seven modules had 123 hub proteins, of which 102 (83%) were associated with cognitive trajectory (Supplementary Data 8). In BLSA, among the 131 hub proteins in the seven modules, 99 (76%) were associated with cognitive trajectory (Supplementary Data 8). We performed the same analysis, except that the input proteins were those that associate with cognitive trajectory independently of pathologies. We found that among the 123 hub proteins in Banner and 127 hub proteins in BLSA, 67 and 62 respectively were also associated with cognitive trajectory independently of β-amyloid plaques and neurofibrillary tangles (Supplementary Data 8). In common between these 67 (Banner) and 62 (BLSA) hub and cognitive trajectory-associated proteins were 38 proteins (Table 5, Supplementary Data 8). Notably, these 38 proteins were associated with cognitive trajectory independently of β-amyloid plaques and neurofibrillary tangles in both Banner and BLSA, and were also hub proteins in both Banner and BLSA and thus are tractable targets for further mechanistic studies of cognitive stability.

**Table 5:** Co-expression hub proteins associated with cognitive trajectory

Protein	Function	Effect size	Adjusted *P*-value	Direction
DLG4|P78352-3	Synaptic function	1.6554	0.00016497	++
SYT1|P21579	Synaptic function	1.5316	0.00026653	++
HOMER1|Q86YM7	Synaptic function	1.2889	0.00040777	++
BAIAP2|Q9UQB8-3	Synaptic function	1.5948	0.00042608	++
ATP6V0D1|P61421	Synaptic function	1.4719	0.0004631	++
SLC30A3|Q99726	Synaptic function	0.6046	0.00090009	++
GRIA2|P42262	Synaptic function	1.3181	0.00174731	++
DMXL2|Q8TDJ6	Synaptic function	1.5595	0.00214875	++
SLC17A7|Q9P2U7	Synaptic function	1.1304	0.00419658	++
ATP6V0A1|Q93050-1	Synaptic function	1.3017	0.00520941	++
SYNPO|Q8N3V7-3	Synaptic function	0.5984	0.0054183	++
ANKS1B|Q7Z6G8-3	Synaptic function	0.9659	0.00597171	++
AP2B1|P63010	Synaptic function	1.7094	0.01001969	++
SEPT11|D6RGI3	Synaptic function	1.8515	0.01278615	++
BSN|Q9UPA5	Synaptic function	0.957	0.01474302	++
AP2A2|O94973	Synaptic function	1.5492	0.01679196	++
STX1A|Q16623	Synaptic function	0.9072	0.02188469	++
AP2A1|O95782-2	Synaptic function	1.6634	0.03793744	++
HSPA2|P54652	Myelination	−0.822	0.00140989	−−
NEFL|P07196	Myelination	−0.5826	0.00746409	−−
NEFM|E7ESP9	Myelination	−0.5688	0.01161255	−−
MBP|P02686	Myelination	−0.3966	0.01545858	−−
CNP|P09543	Myelination	−0.4468	0.02383309	−−
PDHA1|P08559	Mitochondrial function	1.7283	0.00020668	++
TUFM|P49411	Mitochondrial function	1.7968	0.00346194	++
NDUFV1|P49821-2	Mitochondrial function	1.2578	0.0036826	++
NNT|Q13423	Mitochondrial function	1.7308	0.00433757	++
NDUFS1|P28331	Mitochondrial function	1.2152	0.00693232	++
NDUFS2|O75306	Mitochondrial function	1.1988	0.00815356	++
NDUFA10|A0A087WXC5	Mitochondrial function	1.2738	0.01359488	++
NDUFA9|Q16795	Mitochondrial function	1.0601	0.02221231	++
UQCRFS1|P47985	Mitochondrial function	1.076	0.02672126	++
ACO2|Q99798	Mitochondrial function	1.3845	0.04333576	++
NDUFS5|O43920	Mitochondrial function	0.876	0.04357237	++
LLGL1|Q15334	Catabolic process and apoptosis	−0.7602	0.02131939	−−
GSN|P06396-2	Catabolic process and apoptosis	−0.6694	0.02687423	−−
GFAP|P14136	Catabolic process and apoptosis	−0.3761	0.03997317	−−
LMNA|P02545	Catabolic process and apoptosis	−0.7264	0.04584879	−−

This table summarizes 38 hub proteins that were associated with cognitive trajectory after adjusting for β-amyloid plaques and neurofibrillary tangles and their Pearson correlation with cognitive trajectory. The *p*-values are from the meta-analysis of the proteome-wide association studies of cognitive trajectory adjusting for β-amyloid plaques and neurofibrillary tangles from Supplementary Data 5. The underlying data are available through the Synapse platform (https://www.synapse.org/#!Synapse:syn7170616 and https://www.synapse.org/#!Synapse:syn3606086) and Supplementary Data 1

Remarkably, the top protein of this 38-protein list is DLG4, also known as PSD95, which is one of the most abundant scaffold proteins at the excitatory brain synapses and known to affect brain development, neuronal plasticity, memory and learning, and overall cognitive function^24–28^. We asked whether proteins known to interact with PSD95, i.e., PSD95 interactome, were also associated with cognitive trajectory using the published PSD95 interactome from Fernandez et al.^24^. Among the 118 proteins known to interact with PSD95, 109 were detected in the Banner cohort. The first principal component of these 109 proteins, which captures 99.3% of the variance of the PSD95 interactome, was significantly associated with cognitive trajectory by linear regression (*β* = 0.016; *p* = 0.0001; *N* = 104; Supplementary Fig. 6). This implies that higher expression of the PSD95 interacting partners was associated with cognitive stability (Supplementary Fig. 6).

## Discussion

We performed an unbiased proteome-wide association study of cognitive trajectory in a discovery and replication cohort, followed by a meta-analysis, to identify biological processes underlying cognitive stability. Particularly, we used both proteome-wide differential analysis and protein co-expression network analysis to gain insights into changes in individual proteins as well as networks of proteins in cognitive trajectory. Our most notable finding is that proteins involving mitochondrial activities or synaptic functions had increased abundance among individuals with cognitive stability regardless of the burden of β-amyloid plaques or neurofibrillary tangles. In other words, cognitive stability is associated with increased mitochondrial and synaptic activities independently of the burden of β-amyloid plaques or neurofibrillary tangles in neurons predominantly. These findings were derived from two approaches in two different cohorts: (i) proteome-wide association study of cognitive trajectory followed by GO enrichment analysis of the associated proteins, and (ii) protein co-expression network enrichment analysis of the cognitive trajectory-associated proteins. Most interestingly, mitochondrial activities are strikingly present in our analysis in a way not seen when considering the clinical diagnosis of cognitive status or AD pathologic outcomes. For instance, BLSA module M3, enriched for mitochondrial function, was most significantly associated with cognitive trajectory both before and after adjusting for plaques and tangles. This module was not significantly associated with clinical diagnosis or AD pathologies after multiple testing correction in our previous publication^23^, suggesting that cognitive trajectory captures biological processes that are independent of traditional AD pathologies.

Mitochondria in neurons are crucial for maintaining synaptic function, and most of the mitochondrial proteins are encoded in the nuclear genome^29^. In the brain, mitochondria regulate synaptic transmission by generating ATP to power the process and by modulating presynaptic calcium level, which in turn determines the release of neurotransmitters^29^. Synaptic mitochondria, therefore, are vital for the maintenance of synaptic function and transmission^29–31^. This is consistent with our finding that 157 proteins of the 579 cognitive trajectory-associated proteins are mitochondrial proteins and of these, 122 are located in the mitochondria either in the pre- or postsynaptic density. Mitochondrial dysfunction has been suggested to occur early in the progression to AD and continues into later stages of AD^31–34^. An example of early manifestation of mitochondrial dysfunction is a study of expired young-adult *APOE4* carriers vs. expired young-adult non-*APOE4* carriers^32^. In that study, young-adult participants in both groups did not have amyloid or tangle deposition in the posterior cingulate cortex (PCC); yet, *APOE4* carriers, i.e., those at greater risk for developing AD, showed reduced mitochondrial activity in PCC neurons, as measured by cytochrome oxidase levels, suggesting that a change in mitochondrial function may be the earliest manifestation of AD risk^32^. An example of mitochondrial dysfunction continuing into later stages of AD is a transcriptomic study of post-mortem brain of AD cases and controls in the PCC and other brain regions^33^. This study found that the nuclear genes encoding the mitochondrial electron transport chain subunits had decreased expression in AD cases compared to controls in PCC and other brain regions^33^. Furthermore, a transgenic mouse model showed that amyloid pathology, even at a low level, can induce deficits in the functioning of synaptic mitochondria leading to synaptic degeneration^30^. Synaptic degeneration has been suggested to be one of the earliest cellular event in AD pathogenesis, and synaptic loss to be the best correlate of cognitive impairment in AD (see review by Reddy et al.^31^). These findings are consistent with ours. Here, we show proteins involved in mitochondrial activities had lower-abundance in persons with faster cognitive decline but higher-abundance in persons with cognitive stability in an unbiased human proteome-wide study. Taken together, our findings and others highlight that mitochondrial activities would be a fruitful research target for early prevention of cognitive decline and enhancement of cognitive stability.

Synaptic function in cognitive decline has been given more attention than the mitochondrial function. For instance, a study of candidate synaptic proteins in cognitive decline in AD found that reduced abundance of the synaptic protein SNAP25 was associated with faster cognitive decline^35^. Consistently, in both Banner and BLSA, we found that lower-abundance of SNAP25 was associated with faster cognitive decline. Furthermore, Bereczki and colleagues performed a comprehensive study of 851 synaptic proteins in neurodegenerative diseases and cognitive decline^36^. They found that reduced levels of synaptic proteins SNAP47, SYBU, LRFN2, SV2C, and GRIA3 were associated with cognitive impairment and faster cognitive decline^36^. Among these proteins, only GRIA3 was detected in our datasets and had lower-abundance in faster cognitive decline in both Banner and BLSA, consistent with Bereczki and colleagues’ finding.

The next notable finding is the 38 proteins that were associated with cognitive trajectory independently of typical AD neuropathologies and were also hub proteins in both Banner and BLSA protein networks. These proteins are highly promising targets for future mechanistic studies of cognitive trajectory. Among these 38 proteins, those that are higher-abundance in cognitive stability are involved in either mitochondrial activities or synaptic function, and those with lower-abundance in cognitive stability are involved in myelination or apoptosis. Among the proteins involved in synaptic function is DLG4, also known as PSD-95 (postsynaptic density-95 protein). PSD-95 is a major scaffold protein of the dendritic spines and its expression level has been shown to be altered in aging, AD, and several psychiatric disorders^37^. PSD-95 is important for neuronal plasticity and memory consolidation via its regulation of synaptic glutamate receptors, signaling proteins, adhesion molecules, and cytoskeletal proteins^37,38^. Here we show that the aggregate of PSD-95 interactome abundance is also associated with cognitive trajectory. Bustos and colleagues showed that increased protein level of PSD95 in the hippocampus rescued memory deficits of aged mice and dementia mice^38^. Their finding is consistent with ours in which overexpression of PSD95 protein was associated with cognitive stability. The second protein on the list, SYT1, was found to have reduced abundance in AD^39^. Overexpression of Syt1 in mouse hippocampi was found to promote protective presenilin-1 conformation^39^, consistent with our finding of its protective effect. Another protein on this list, HOMER1 (i.e., HOMER1a) is an important protein for synaptic plasticity and memory consolidation because it drives homeostatic scaling-down of excitatory synapses during sleep to remodel synapses and consolidate contextual memory^40^. Consistently, we found that the protein level of HOMER1 was increased in cognitive stability. SLC30A3, aka ZNT3, is a synapse-specific vesicular zinc transporter that is important for zinc homeostasis and required for hippocampus-dependent memory^41^. Consistently, we found that SLC30A3 protein level was increased in cognitive stability. SLC17A7, aka VGLUT1, is a sodium-dependent phosphate transporter that is specifically expressed in the neuron-rich regions of the brain and transports glutamate. Decreased abundance of this protein can lead to susceptibility to neuroinflammation and disruption of synaptic plasticity^42^. Along the same theme, SYNPO has been found to be important for synaptic plasticity^43,44^. A notable theme among the proteins participating in synaptic function on this list of 38 proteins is that they are important for neuronal plasticity and memory consolidation and their overexpression is associated with cognitive stability.

Some proteins, including NEFM and MBP, participate in myelination on this list of 38 proteins. Myelination refers to the formation of a membranous sheath surrounding axons to increase signal transmitting speed between neurons and is not limited to early development but occurs throughout adulthood^45^. NEFM are neurofilaments that maintain neuronal caliber and participate in intracellular transport to axons and dendrites. NEFM was shown to accumulate in the hippocampus in the presence of amyloid pathology^46^. To retard human aging and age-related diseases, inhibition of insulin-like growth factor 1 (IGF1) has been proposed, and several studies have shown that neuron-specific deletion of IGF1R confers neuroprotection and improves behavior in AD transgenic mice^46–48^. From a genome-wide screen, a prominent observation after genetic ablation of IGF1 receptor in AD mice is the reduction in expression level of NEFM, down to control level, suggesting that lower-abundance of NEFM is protective^46^. These findings are consistent with ours, in which lower-abundance of NEFM was associated with cognitive resilience. Myelin basic protein, MBP, can influence β-amyloid accumulation in the brain. Indeed, deletion of MBP gene led to a significant reduction in cerebral β-amyloid levels in Tg-3xFAD mice^49^, consistent with our finding of decreased abundance of MBP being associated with cognitive stability.

Besides the 38 proteins above, we also presented (i) 579 proteins associated with cognitive trajectory from our meta-analysis, (ii) 232 proteins associated with cognitive trajectory independently of amyloid plaques and neurofibrillary tangles, (iii) 102 proteins and 99 proteins associated with cognitive trajectory and were also hub proteins in Banner networks and BLSA networks, respectively. Furthermore, we note that VGF is strongly associated with cognitive trajectory with or without adjusting for AD neuropathology. VGF is also a hub protein for Banner M1 module, which involves synaptic function. Our findings suggest that VGF likely act through mechanisms independent of β-amyloid plaques and neurofibrillary tangles in contributing to cognitive decline. VGF is a neuropeptide that promotes hippocampal neurogenesis, dendritic maturation, and synaptic activity^50^. VGF is induced by BDNF and serotonin and is upregulated by antidepressants, exercise, and is reduced in animal models of depression^51^. VGF is another promising candidate for the mechanistic study of cognitive stability, consistent with findings from this multiscale causal network modeling of AD^52^.

From the protein co-expression network analysis, we found 10 Banner protein modules and 1 BLSA protein module associated with cognitive trajectory. Fewer networks remained associated with cognitive trajectory after adjusting for β-amyloid plaques and neurofibrillary tangles, suggesting that some networks act through pathology while others act independently of pathology to contribute to variation in cognitive trajectory. We also note that the published BLSA protein co-expression modules used here were constructed using the protein expression levels from both the dorsolateral prefrontal cortex (dPFC) and precuneus so the module memberships might be slightly different than those from the dPFC alone. On the other hand, proteins and protein co-expression networks that are consistently changed within the two regions likely reflect biologically meaningful differences.

We found cognitive trajectory-associated proteins to be enriched in astrocytes, which perform important roles for neural functioning. Astrocytes provide neurotrophic support to promote neuronal survival, are important in the formation and maturation of synapses, help control neurotransmitter concentrations, and play a role in maintaining the blood-brain barrier^53^. We found that both higher- and lower-abundance proteins in cognitive stability were enriched in astrocytes. Our findings may reflect the two types of astrocytes as described in the literature—reactive astrocytes that lose the ability to promote neuronal survival, outgrowth, and synaptogenesis, and induce death of neurons and oligodendrocytes, and homeostatic astrocytes that promote neural functioning^54^.

Our findings should be interpreted in light of the strengths and limitations of this study. First, this is an association study and thus it precludes causal inference. However, we nominated the tractable targets for further mechanistic studies of cognitive trajectory. Second, our label-free proteomic method yielded approximately 3900 proteins in the proteome, which is not as comprehensive of a proteomic profile as would be ideal, and larger proteomic analysis will undoubtedly add refinement to the proteins and protein networks involved in cognitive trajectory. Third, the available cognitive data was from the MMSE, a widely used screening tool that provides a coarse gauge of cognitive performance. Nevertheless, the decline in MMSE generally parallels that of more detailed testing^55^. Fourth, our sample sizes are relatively small (*n* = 104 and *n* = 39) and exclusively Caucasians, which may limit the generalizability of our findings and power. Despite this, ours is the largest brain proteomic study of cognitive trajectory to our knowledge. These subjects were followed for a total of 887 person-years over 676 visits. We also employed a discovery and replication cohort design followed by meta-analysis to reduce false positive findings. Furthermore, our focus on individual cognitive trajectory is more germane to early intervention and precision medicine. Finally, our findings establish a framework for understanding mechanisms underlying cognitive trajectory and nominate a list of highly promising targets for future mechanistic studies of cognitive stability.

## Methods

### Banner sun health research institute participants

This project, the Arizona Study of Aging and Neurodegenerative Disorders, is a longitudinal clinicopathological study of normal aging, Alzheimer’s disease (AD), and Parkinson’s disease (PD)^56^. Most subjects were enrolled as cognitively unimpaired volunteers from the retirement communities of the greater Phoenix, Arizona, USA^56^. Recruitment efforts were also directed at subjects with AD and PD from the community and neurologists’ offices. Subjects received standardized general medical, neurological, and neuropsychological tests annually during life and more than 90% received full pathological examinations after death^56^. For this study, we only included subjects with a clinical diagnosis of AD or normal control rendered approximate to death. Please refer to Table 1 for subject characteristics. Additional inclusion criteria for this study were having a baseline score from the Mini Mental State Examination (MMSE) of 27 or above (i.e., non-demented at baseline)^57,58^ and at least one additional MMSE score in subsequent follow-up visits. All enrolled subjects in the Banner dataset or their legal representatives sign a Banner Sun Health Research Institute Institutional Review Board-approved informed consent form allowing both clinical assessments during life, several options for brain and/or bodily organ donation after death, and usage of donated biospecimens for approved future research^56^.

### Baltimore Longitudinal Study of Aging (BLSA) participants

BLSA is a prospective cohort study of aging in community-dwelling individuals^59,60^. It continuously recruits healthy volunteers aged 20 or older and follow them for life regardless of changes in health or functional status. Participants were examined at the National Institute of Aging Clinical Research Unit in Baltimore at one to four-year intervals, with more frequent follow-up visits for older participants^59,60^. Due to a smaller sample size and the fact that these subjects were recruited as cognitively unimpaired individuals, all BLSA individuals with proteomic data were included regardless of MMSE score at baseline. The BLSA study was approved by the Institutional Review Board and the National Institute on Aging. Human research at the National Institutes of Health (NIH) is implemented in accord with the U.S. Department of Health and Human Services (45 CFR46) and U.S. Food and Drug Administration (21 CFR 50 and 56) regulations for the protection of human subjects. The NIA IRB is part of the Human Subject Protection Program of the NIH. All BLSA participants provided written informed consent at each visit^61^.

### Personalized cognitive trajectory

Person-specific cognitive trajectory was estimated using a linear mixed model for each subject. For each cohort, we model the annual MMSE score as a longitudinal outcome, follow-up year as the independent variable and sex, age at enrollment, and education as the covariates, with a random intercept and random slope per subject using the lme4 R package (version 1.1-19). The derived cognitive trajectory for each individual in Banner and BLSA cohorts is listed in Supplementary Data 1.

### Pathological assessment of Banner and BLSA cohorts

Measures of AD pathology, i.e., β-amyloid plaques and neurofibrillary tangles, were used as covariates to adjust for the degree of AD pathology in each sample. Beach et al. described in detail how these measures were derived for the Banner cohort^56^. Briefly, plaque total is the summary density score of all types of amyloid plaques in the frontal, temporal, and parietal cortices, as well as hippocampus CA1 region and entorhinal region. Additionally, neuritic plaque density scoring was done according to the Consortium to Establish a Registry for Alzheimer’s Disease (CERAD)^58^. Tangle total is the sum of neurofibrillary tangle density in the frontal cortex, temporal cortex, parietal cortex, hippocampus, and entorhinal region. Tangle scoring was done according to the CERAD templates^58^. In the BLSA cohort, O'Brien et al. described how estimates of β-amyloid positive plaques and neurofibrillary tangles were derived^62^. Briefly, silver staining was performed to assess the severity of neuritic plaques using CERAD stage (range: 0–3) and of neurofibrillary tangles using Braak stage (range: 1–6), with higher scores indicating higher level of severity.

### Proteomic measurements

Proteomic quantification for both cohorts used the approach described in detail in Seyfried et al.^23^ and enumerated below. For both cohorts, whole-brain proteomes were derived from cortically dissected dorsolateral prefrontal cortex for the discovery and replication cohorts, separately. Approximately 100 mg of tissue was combined with 500 μL of urea lysis buffer (8 M urea, 100 mM NaHPO4, pH 8.5) and 5 μL (100× stock) HALT protease and phosphatase inhibitor cocktail (Pierce) in a 1.5 mL Rino tube (Next Advance) with a 750 mg stainless steel bead (0.9–2 mm in diameter). Homogenization was performed using a Bullet Blender (Next Advance) at 4 °C with two 5-min intervals of blending. Supernatant was transferred to 1.5 mL Eppendorf tube and sonicated (Sonic Dismembrator, Fisher Scientific) 3 times for 5 s with 15 s intervals of rest at 30% amplitude to disrupt nucleic acids and subsequently vortexed. Protein concentration was determined by the bicinchoninic acid (BCA) method, and samples were frozen in aliquots at −80 °C. Each homogenate was analyzed by SDS-PAGE to assess for protein integrity. Protein homogenates (150 μg) were diluted with 50 mM NH_4_HCO_3_ to a final concentration of less than 2 M urea and then treated with 1 mM dithiothreitol (DTT) at 25 °C for 30 min, followed by 5 mM iodoacetimide (IAA) at 25 °C for 30 min in the dark. Protein was digested with 1:100 (w/w) lysyl endopeptidase (Wako) at 25 °C for 2 h and further digested overnight with 1:50 (w/w) trypsin (Promega) at 25 °C. Resulting peptides were desalted with a Sep-Pak C18 column (Waters) and dried under vacuum. For quantification, peptides (2 μg) were resuspended in peptide loading buffer (0.1% formic acid, 0.03% trifluoroacetic acid, 1% acetonitrile) containing 0.2 pmol of isotopically labeled peptide calibrants (Life Technologies, #88321). Peptide mixtures were separated on a self-packed C18 (1.9 um Dr. Maisch, Germany) fused silica column (25 cm × 75 uM internal diameter (ID); New Objective, Woburn, MA) by a NanoAcquity UHPLC (Waters, Milford, FA) and monitored on a Q-Exactive Plus mass spectrometer (ThermoFisher Scientific, San Jose, CA). For the Banner cohort, elution was performed over a 120-min gradient at a rate of 275 nL/min with buffer B ranging from 3 to 60% (buffer A: 0.1% formic acid and 5% DMSO in water, buffer B: 0.1% formic and 5% DMSO in acetonitrile). For the BLSA cohort, elution was also performed over a 120-min gradient but at a rate of 250 nL/min with Buffer B ranging from 3 to 80% (buffer A: 0.1% formic acid in water, buffer B: 0.1% formic acetonitrile). For both cohorts, the mass spectrometer cycle was programmed to collect one full MS scan followed by 10 data-dependent MS/MS scans. The MS scans (300–1800 *m/z* range,1,000,000 AGC, 100 ms maximum ion time) were collected at a resolution of 70,000 at *m/z* 200 in profile mode and the MS/MS spectra (2 *m/z* isolation width with 0.5 *m/z* offset, 30% collision energy, 50,000 AGC target, 50 ms maximum ion time) were acquired at a resolution of 17,500 at *m/z* 200. Dynamic exclusion was set to exclude previously sequenced precursor ions for 30 s within a 10 ppm window. Precursor ions with +1, and +6 or higher charge states were excluded from sequencing.

MaxQuant (v1.5.3.30 for Banner cohort and v1.5.2.8 for BLSA cohort) with Thermo Foundation 2.0 for RAW file reading capability was used to generate label-free quantification. The search engine Andromeda, integrated into MaxQuant 1, was used to build and search a concatenated target-decoy IPI/Uniprot human reference protein database (retrieved April 20, 2015; 90,411 target sequences), plus 245 contaminant proteins from the common repository of adventitious proteins (cRAP) built into MaxQuant. Methionine oxidation (+15.9949 Da), asparagine and glutamine deamidation (+0.9840 Da), and protein N-terminal acetylation (+42.0106 Da) were variable modifications (up to 5 allowed per peptide); cysteine was assigned a fixed carbamidomethyl modification (+57.0215 Da). Only fully tryptic peptides were considered with up to 2 miscleavages in the database search. A precursor mass tolerance of ± 20 ppm was applied prior to mass accuracy calibration and ± 4.5 ppm after internal MaxQuant calibration. Other search settings included a maximum peptide mass of 6000 Da, a minimum peptide length of 6 residues, 0.05 Da tolerance for high resolution MS/MS scans. Co-fragmented peptide search was enabled to deconvolute multiplex spectra. The false discovery rate (FDR) for peptide spectral matches, proteins, and site decoy fraction were all set to 1 percent. Quantification settings were as follows: requantify with a second peak finding attempt after protein identification has completed; match MS1 peaks between runs; a 0.7 min retention time match window was used after an alignment function was found with a 20-min RT search space. The quantitation method only considered razor plus unique peptides for protein level quantitation. Options to access the full list of parameters used for MaxQuant, raw and searched proteomic data are described in the Data Availability section. In our prior work on the BLSA dataset, we used data from the dorsolateral prefrontal cortex (dPFC) and precuneus regions together; however, in this work, we only focused on proteins from the dPFC. After label-free quantification, 3710 protein groups in Banner and 3933 protein groups in BLSA cohorts were detected, and 2752 protein isoforms were detected in both Banner and BLSA cohorts.

### Proteomic quality control

Only proteins quantified in at least 90% of the samples were included in the analysis. We performed log_2_ transformation of the proteomic profiles. Subsequently, linear mixed models from the R package variancePartition^63^ were used to characterize the percent of protein variance explained by biological and technical variables. This approach was used to quantify the main sources of variation in each proteomic dataset separately and identified the variance attributable to age, sex, neuropathology, post-mortem interval (PMI), and batch. For instance, in the Banner cohort, batch and sex explained substantial proportions while age at death and PMI explained some proportions of variance of the proteomic profile (Supplementary Fig. 2). Therefore, within each cohort, effect of batch was removed using Combat^64^, and effects of sex, age at death, and PMI were removed using bootstrap regression. We removed these technical (batch and PMI) and biological effects (sex and age at death) because they may confound our association analyses between cognitive trajectory and protein levels. Supplementary Fig. 3 shows that after Combat and bootstrap regression, batch, sex, age at death, and PMI explained only minimal amounts of the variance of the proteomic profile. The same approach was used to successfully remove the effects of these same covariates from the BLSA proteome^23^.

### Proteome-wide association study of cognitive trajectory

Proteome-wide association analysis was performed in each cohort separately, and meta-analysis was used to combine individual results. In each cohort, linear regression was performed with cognitive trajectory as the outcome and normalized protein abundance as the predictor. In our planned secondary analysis, we performed the same association analysis as above but adding β-amyloid plaques and neurofibrillary tangles as covariates. Meta-analysis was performed with METAL^65^, a popular meta-analysis tool^66^, using effect size estimates and standard errors from the proteome-wide association studies of cognitive trajectory in Banner and BLSA, respectively. We included proteins that are detected in both the Banner and BLSA cohorts in the meta-analysis. For all analyses, we used Benjamini–Hochberg (BH) method to control the false discovery rate (FDR)^67^, and declared significantly associated proteins as those with BH FDR *p* < 0.05.

### Protein co-expression network analysis

Network analysis was performed in Banner and BLSA datasets separately to identify modules of co-expressed proteins. For the Banner network, missing proteins were imputed using the k-nearest neighbor imputation function in R. Then, batch effects were removed using Combat^64^, and age at death, sex, and PMI were regressed from the proteomic profiles using Bootstrap regression. Weighted gene co-expression network analysis (WGCNA)^68^ was used on normalized protein abundance to define protein co-expression networks. For BLSA, we used the BLSA networks from Seyfried et al.^23^, which were previously built using proteins measured from the precuneus and prefrontal cortex in the same individual. We defined hub proteins, i.e., highly connected proteins, for each of the modules as those with intramodular kME in the top 90th percentile among the proteins in the corresponding module^68^. Gene ontology (GO) enrichment analysis was performed on each protein co-expression module using GO Elite and Fisher exact test^69^ to glean a deeper biological understanding of these modules.

### Enrichment analysis of cognitive trajectory proteins

GO enrichment analysis was performed on the proteins associated with cognitive trajectory at FDR < 0.05 from the meta-analysis. Proteins were categorized into two lists for GO enrichment analysis: (i) higher-abundance in cognitive stability, and (ii) lower-abundance in cognitive stability. Gene sets were downloaded from MSigDB (version 2017), including GO biological process, cellular component, and molecular function. We also downloaded the cell type-specific protein expression in the brain generated by Sharma et al.^19^, as well as cell type-specific gene expression data generated by Darmanis et al.^21^ and Zeisel et al.^20^ to assess for enrichment of the significantly associated proteins/genes in particular brain cell types. For enrichment analysis, we used a modified version of the GeneOverlap function (from package of the same name) in R and Fisher’s exact test so that all pair-wise tests were adjusted for multiple testing using BH method to control the FDR^67^. The Fisher’s test function also provides an estimated odds-ratio in comparison to a proteomic background set to 20,000.

### Reporting summary

Further information on experimental design is available in the Nature Research Reporting Summary linked to this article.

## Supplementary information

Supplementary Information

Description of Additional Supplementary Files

Supplementary Data 1

Supplementary Data 2

Supplementary Data 3

Supplementary Data 4

Supplementary Data 5

Supplementary Data 6

Supplementary Data 7

Supplementary Data 8

Reporting Summary

Source Data

## Data Availability

All proteomic and phenotypic data that support the findings of this study are contained in Supplementary Data 1 and in https://www.synapse.org/#!Synapse:syn7170616 and https://www.synapse.org/#!Synapse:syn3606086. Source data underlying Fig. 5 are provided as a Source Data file. A reporting summary for this Article is available as a Supplementary Information file. All other data supporting the findings of this study are available from the corresponding authors on reasonable request.
