# Supplementary material for: Large-scale proteomic analysis of human brain identifies proteins associated with cognitive trajectory in advanced age
Source: Nat Commun. 2019 Apr 8;10:1619. doi: 10.1038/s41467-019-09613-z (PMC6453881; doi:10.1038/s41467-019-09613-z)
Supplement: Supplementary file 1 — Supplementary Information [file 41467_2019_9613_MOESM1_ESM.pdf]

Large-scale proteomic analysis of human brain identifies proteins associated with cognitive trajectory in advanced age

Wingo et al.

Supplementary Figures

Supplementary Figure 1: Volcano plots of proteome-wide association studies of cognitive trajectory.

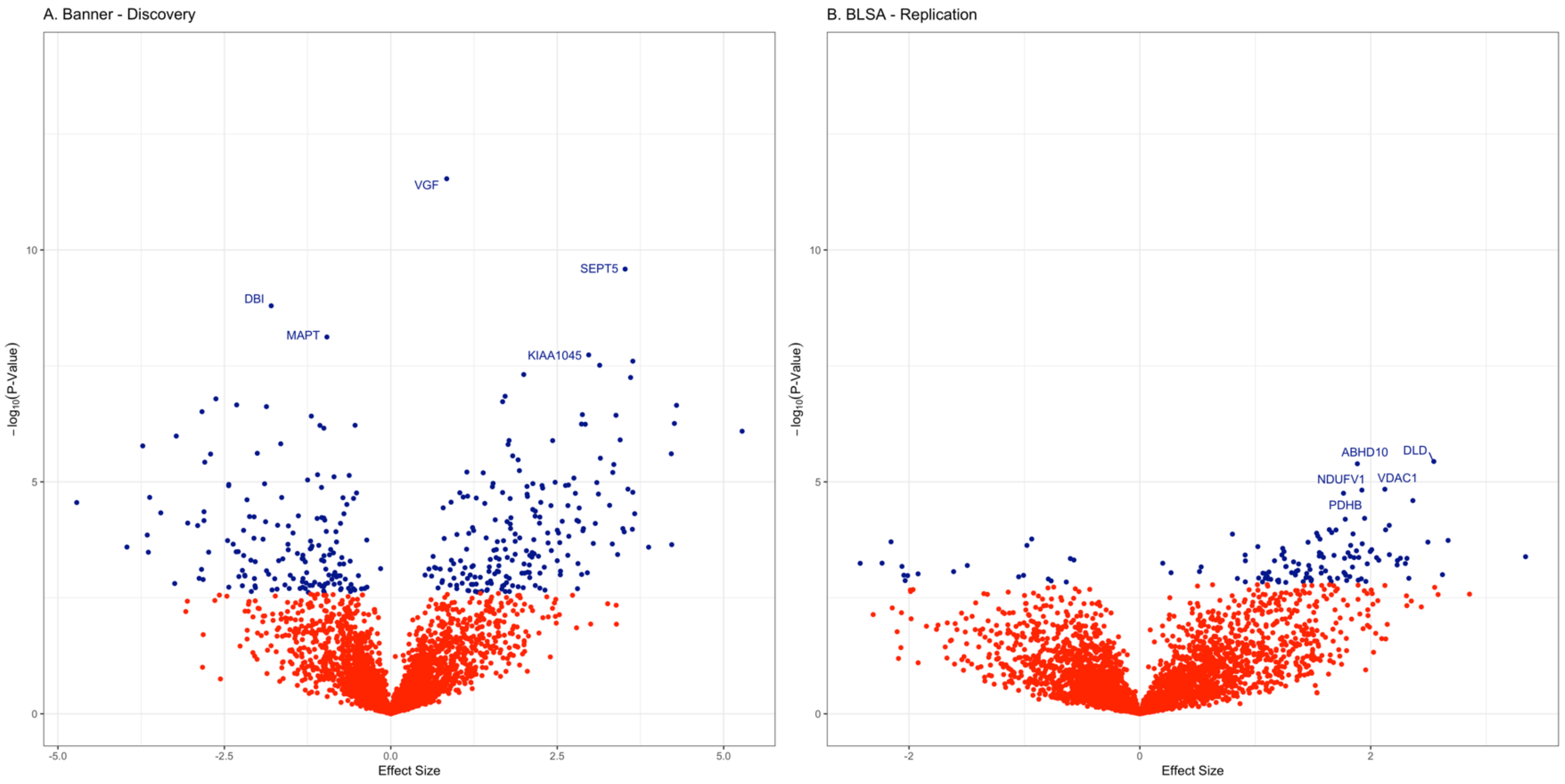

The proteins significantly associated with cognitive trajectory were colored blue. **A.** Banner (Discovery) dataset with the top 5 proteins labeled. **B.** BLSA (Replication) dataset with the top 5 proteins labeled.

Supplementary Figure 2:. Variance partition of Banner proteomic profiles before quality control.

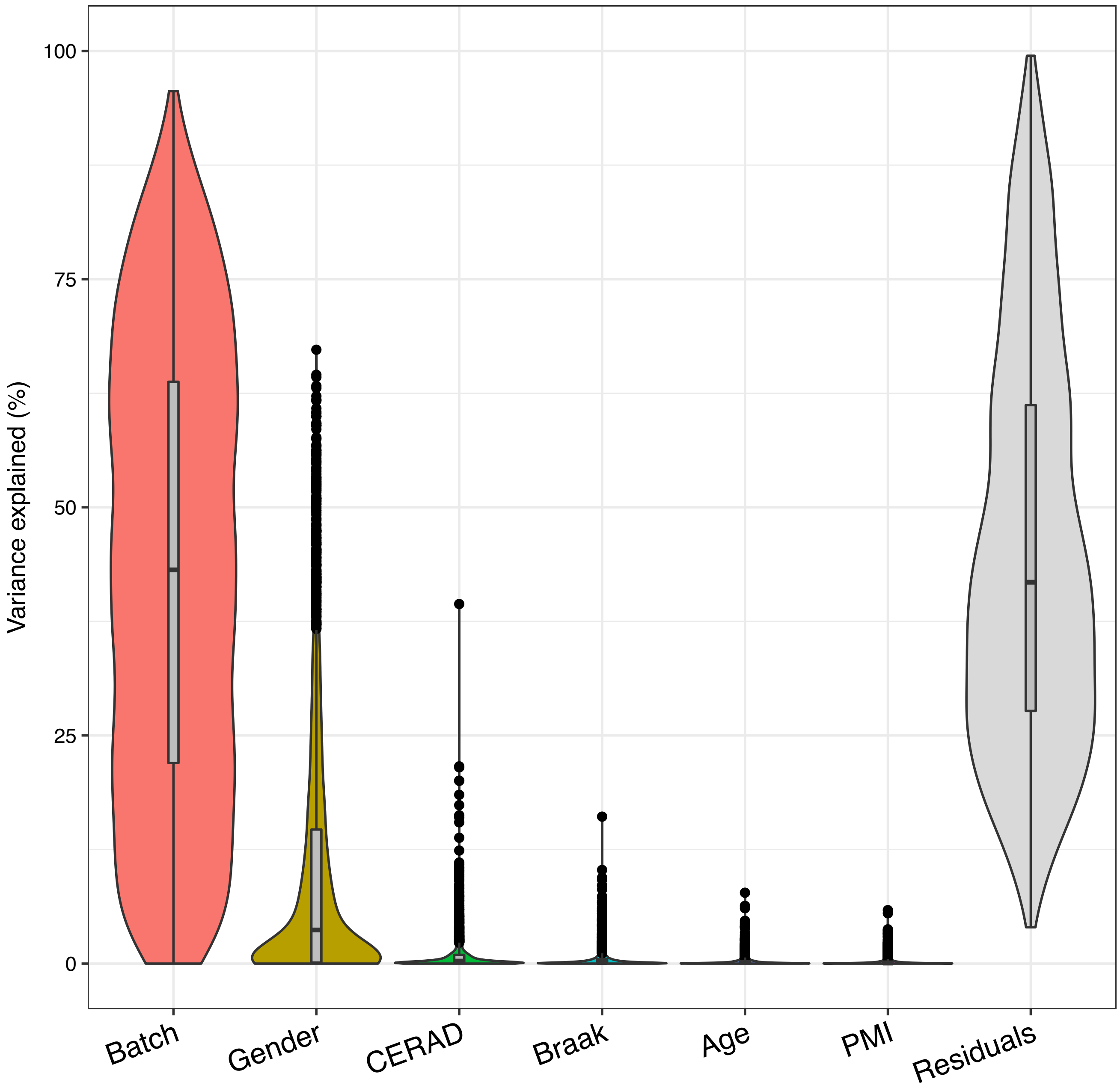

This figure shows the effects (measured by percent variance of proteomic profile) for CERAD Stage, Braak Stage, sex, age at death, post-mortem interval (PMI), and batch prior to quality control. A violin plot is used to summarize the proteome-wide trend and rank the relative contribution of each variable. The outer layer of the violin shows the density of values for all proteins measured, and inside each violin plot, the median percent variance is given as a dark line and the 95% confidence is denoted by a grey box.

Supplementary Figure 3. Variance partition of Banner proteomic profiles after quality control.

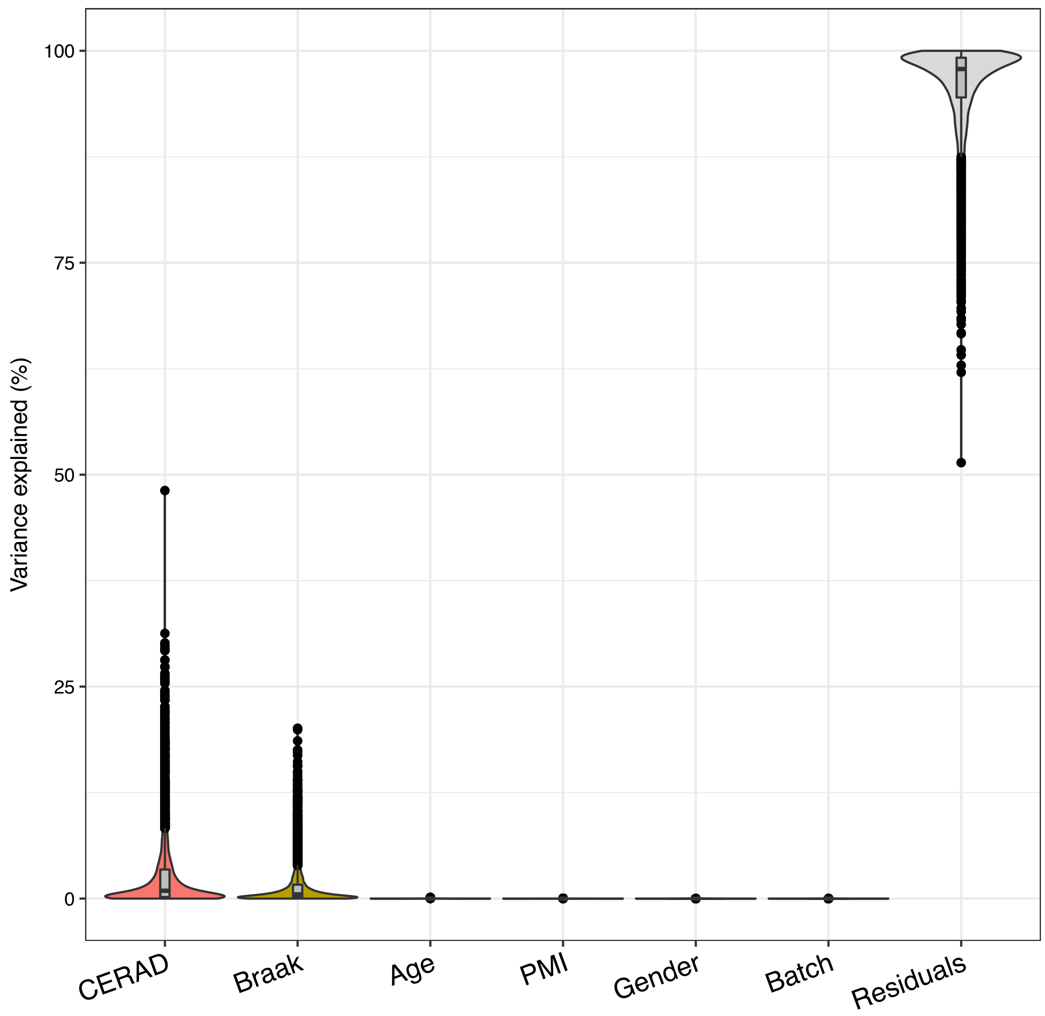

This figure shows successful normalization of the proteomic profile. Percent variance of the proteomic profile is plotted for each covariate: CERAD Stage, Braak Stage, Age at death, post-mortem interval (PMI), sex, and batch after regressing out effects of sex, age at death, PMI, and batch effects as part of the quality control process. A violin plot is used to summarize the proteome-wide trend and rank the relative contribution of each variable. The outer layer of the violin shows the density of values for all proteins measured, and inside each violin plot, the median percent variance is given as a dark line and the 95% confidence is denoted by a grey box.

Supplementary Figure 4. Protein-protein interaction network of cognitive trajectory associated proteins.

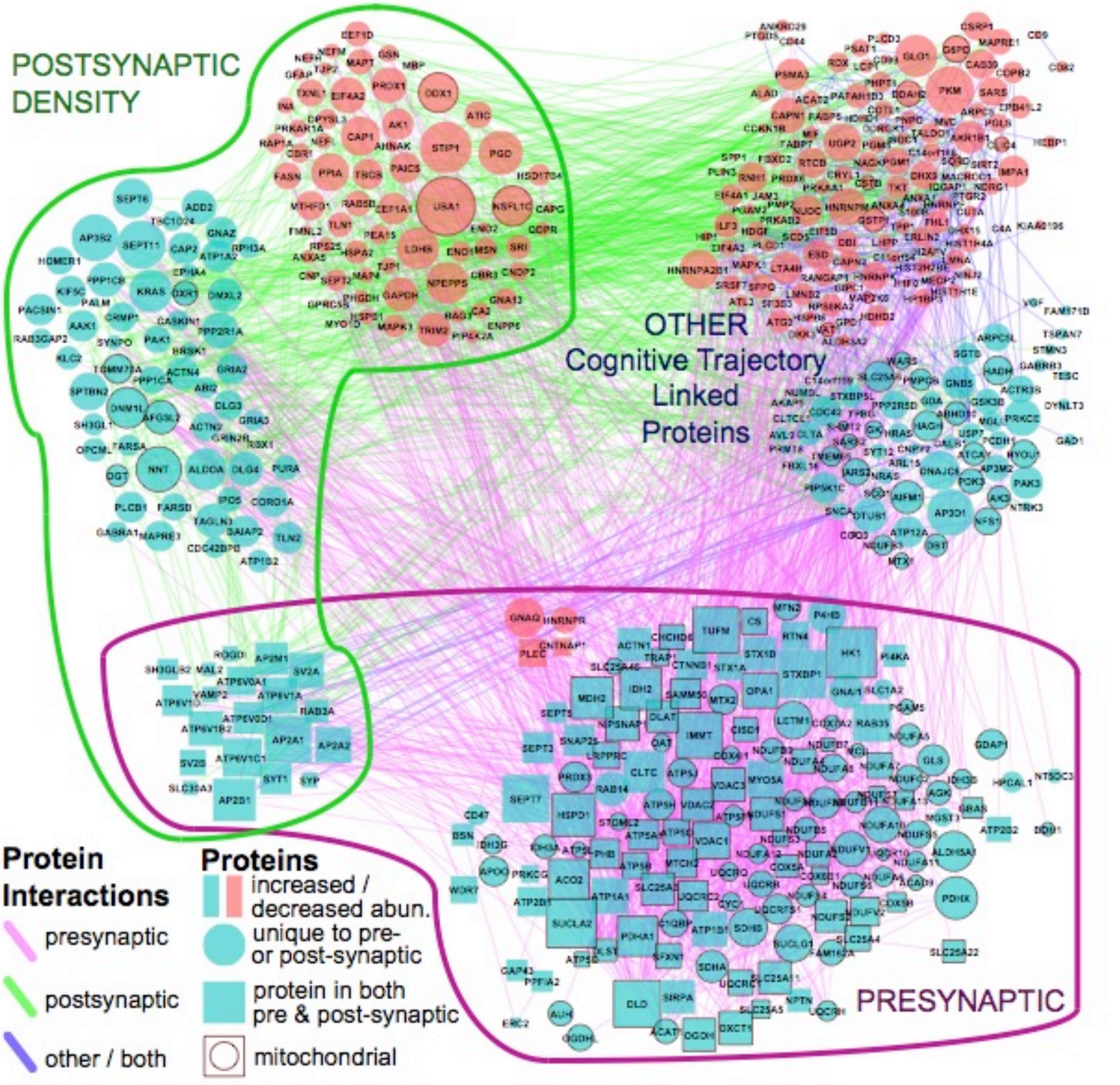

This figure shows a cytoscape protein-protein interaction (PPI) network based on BioGRID among the 579 proteins associated with cognitive trajectory. Among these 579 proteins, 569 were unique proteins. Among these 569 unique proteins, 159 were presynaptic, 150 postsynaptic, 20 present in both pre- and postsynaptic density, and 240 were located in other locations. Additionally, 112 presynaptic proteins, 10 postsynaptic proteins, and 35 other proteins were also designated as mitochondrial proteins.

Supplementary Figure 5: Protein-protein interaction network of cognitive trajectory associated proteins focusing on interactions from pre- or post-synaptic proteins.

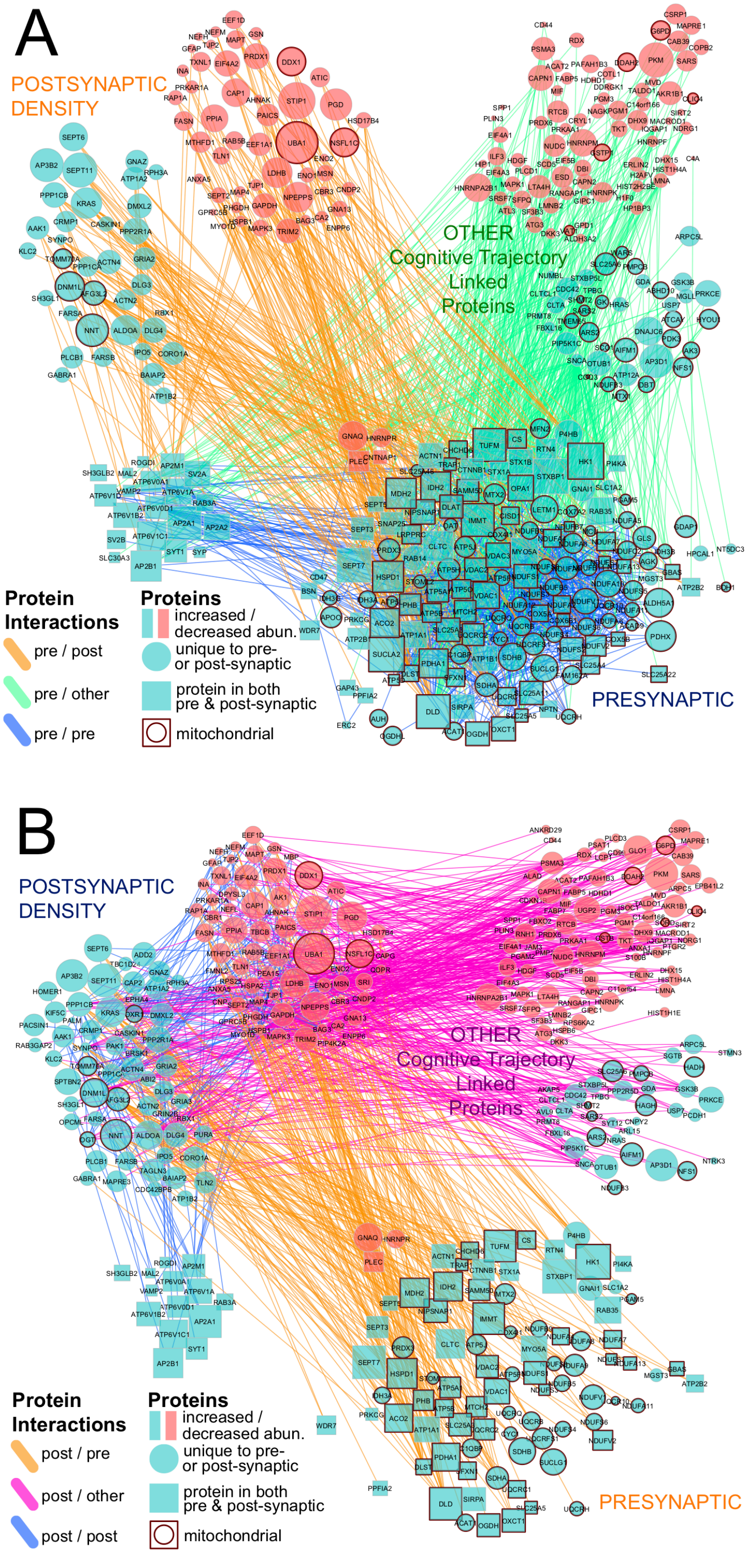

This figure shows a cytoscape PPI network based on BioGRID among the 579 proteins associated with cognitive trajectory. **A)** Focuses on PPIs emanating from presynaptic proteins. **B)** Focuses on PPIs emanating from postsynaptic proteins. Among these 579 proteins, 569 were unique proteins. Among these 569 proteins, 159 were presynaptic, 150 postsynaptic, 20 present in both pre- and postsynaptic density, and 240 proteins were located in other locations. Additionally, 112 presynaptic proteins, 10 postsynaptic proteins, and 35 other proteins were also designated as mitochondrial proteins.

Supplementary Figure 6: Interactome of DLG4/PSD95 and cognitive trajectory in Banner cohort.

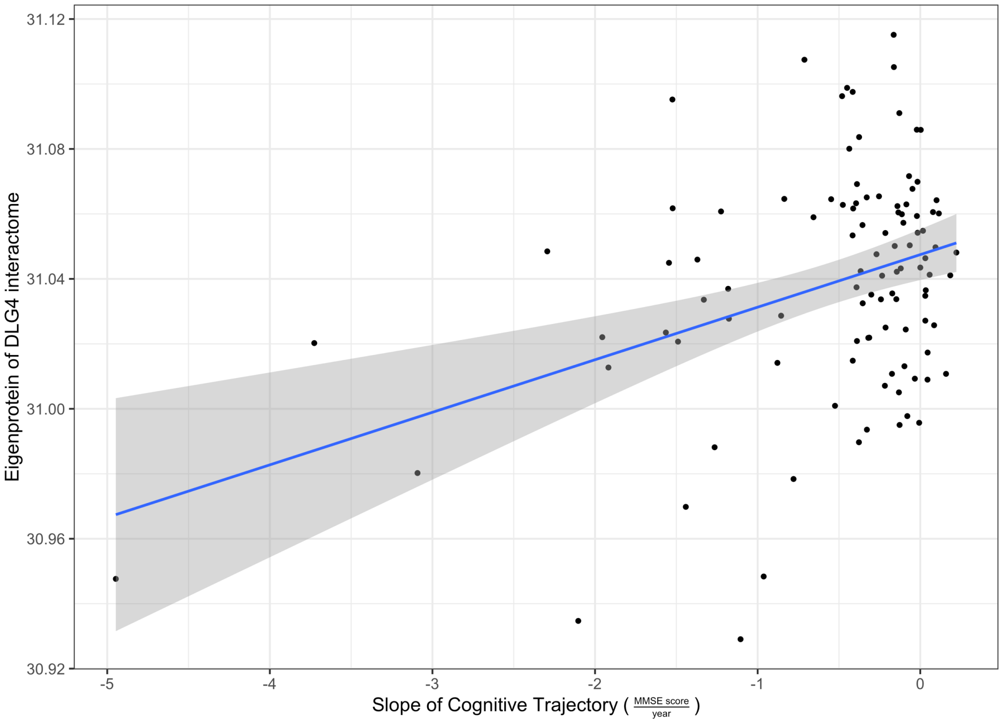

Higher expression of proteins of DLG4/PSD95 interactome was significantly associated with cognitive stability. The grey area denotes 95% confidence interval.
