# Supplementary material for: Large-scale proteomic analysis of human brain identifies proteins associated with cognitive trajectory in advanced age
Source: Nat Commun. 2019 Apr 8;10:1619. doi: 10.1038/s41467-019-09613-z (PMC6453881; doi:10.1038/s41467-019-09613-z)
Supplement: Supplementary file 2 — Description of Additional Supplementary Files [file 41467_2019_9613_MOESM2_ESM.docx]

**Description of Additional Supplementary Files**

**Supplementary Data 1:** Cognitive trajectory data.

**Supplementary Data 2:** Results of proteome-wide association studies of cognitive trajectory.

**Supplementary Data 3:** Cognitive trajectory-associated proteins grouped by localization at either presynaptic, postsynaptic, or both.

**Supplementary Data 4:** Gene ontology enrichment analysis of cognitive trajectory proteins.

**Supplementary Data 5:** Results of proteome-wide association studies of cognitive trajectory adjusted for two measured neuropathologies.

**Supplementary Data 6:** Gene ontology and cell type enrichment analysis of cognitive trajectory-associated proteins adjusted for amyloid and tangles.

**Supplementary Data 7:** Weighted co-expression network analysis in Banner and module overlap between BLSA and Banner co-expression networks.

**Supplementary Data 8:** Cognitive trajectory-associated proteins and their relation to hub proteins from weighted co-expression protein modules.
